# The volume-outcome relationship among severely injured patients admitted to English major trauma centres: a registry study

**DOI:** 10.1186/s13049-020-0710-7

**Published:** 2020-03-06

**Authors:** Charlie A. Sewalt, Eveline J. A. Wiegers, Fiona E. Lecky, Dennis den Hartog, Stephanie C. E. Schuit, Esmee Venema, Hester F. Lingsma

**Affiliations:** 1 Department of Public Health, Erasmus MC University Medical Centre, P.O. Box 2040, 3000 CA Rotterdam, The Netherlands; 2 School of Health and Related Research, Sheffield University. Salford Royal NHS Foundation Trust, Salford, UK; 3 Trauma Audit and Research Network, University of Manchester, Salford, Manchester, UK; 4 Trauma Research Unit, Department of Surgery, Erasmus MC, University Medical Centre, Rotterdam, The Netherlands; 5 Department of Emergency Medicine, Erasmus MC University Medical Centre, Rotterdam, The Netherlands; 6 Department of Internal Medicine, Erasmus MC University Medical Centre, Rotterdam, The Netherlands; 7 Department of Neurology, Erasmus MC University Medical Centre, Rotterdam, The Netherlands

**Keywords:** Volume-outcome relationship, Severely injury, Quality of trauma care

## Abstract

**Background:**

Many countries have centralized and dedicated trauma centres with high volumes of trauma patients. However, the volume-outcome relationship in severely injured patients (Injury Severity Score (ISS) > 15) remains unclear. The aim of this study was to determine the association between hospital volume and outcomes in Major Trauma Centres (MTCs).

**Methods:**

A retrospective observational cohort study was conducted using the Trauma Audit and Research Network (TARN) consisting of all English Major Trauma Centres (MTCs). Severely injured patients (ISS > 15) admitted to a MTC between 2013 and 2016 were included. The effect of hospital volume on outcome was analysed with random effects logistic regression models with a random intercept for centre and was tested for nonlinearity. Primary outcome was in-hospital mortality.

**Results:**

A total of 47,157 severely injured patients from 28 MTCs were included in this study. Hospital volume varied from 69 to 781 severely injured patients per year. There were small between-centre differences in mortality after adjusting for important demographic and injury severity characteristics (adjusted 95% odds ratio range: 0.99–1.01). Hospital volume was found to be linear and not associated with in-hospital mortality (adjusted odds ratio (aOR) 1.02 per 10 patients, 95% confidence interval (CI) 0.68–1.54, *p* = 0.92).

**Conclusions:**

Despite the large variation in volume of the included MTCs, no relationship between hospital volume and outcome of severely injured patients was found. These results suggest that centres with similar structure and processes of care can achieve comparable outcomes in severely injured patients despite the number of severely injured patients they treat.

## Introduction

Injury is the major cause of death in adults younger than 45 years of age [1]. The implementation of trauma systems and dedicated level I trauma centres in the United States has reduced mortality of severely injured patients, usually defined as patients with an Injury Severity Score (ISS) above 15, and improved functional outcome at discharge [2]. In 2012, Regional Trauma Networks with Major Trauma Centre hubs (MTC) were implemented in the English National Health Service - early mortality changes were not found in the immediate post-implementation period [3]. But a recent paper suggests a 19% case fatality reduction over the 5 years since MTC designation [4]. Commissioning and formal designation of Major Trauma Centres was done at national rather than regional level to create uniformity in service provision and equity of access.

Implicit to the centralization of trauma care is the idea that increased volumes of severely injured patients lead to more experienced health care providers, which could result in improved patient outcomes. A recently published systematic review showed that higher hospital volume is associated with lower mortality in severely injured patients [5]. However, hospitals treating severely injured patients do not only differ in hospital volume. Other factors, such as variation in case mix, organization of care, facilities and geographic location could cause between-centre differences. For example, in the field of traumatic brain injury (TBI) considerable between-centre differences have been found, [6–9] but it is still unknown how these are caused. It remains unclear if between-centre outcome differences for severely injured patients exist between major centres and whether they could be explained by differences in hospital volume.

Therefore, the aim of this study was to determine whether there is an association between hospital volume of severely injured patients and patient outcomes in Major Trauma centres (MTCs).

## Patients and methods

### Data

A retrospective observational cohort study was performed using the Trauma Audit and Research Network (TARN) database. TARN is a national trauma registry including all patients with major trauma admitted to hospitals in England and Wales. The TARN includes all patients with significant injury who were admitted for at least 72 h, or to an high-dependency area or who died following arrival at hospital. TARN has UK Health Research Authority Approval (PIAG Section 251) for research on anonymised patient data.

In this study, all patients with an ISS > 15, admitted to an English MTC or transferred to an English MTC between 1 January 2013 and 31 December 2016 were selected from the TARN database. The STROBE statement was used when reporting the data.

### Outcomes

The primary outcome variable was in-hospital mortality. The secondary outcome variables were length of stay (LOS), critical care LOS, time from arrival at the Emergency Department (ED) to first operation and time from arrival at ED to first CT scan.

### Statistical analysis

First, between-centre differences in in-hospital mortality were assessed using a random effects logistic regression model. The first model only contained a random intercept for centre, so the outcome of the patient was only based on the centre that treated the patient. The variance of the random effects was expressed as tau-squared. If tau-squared is above 1, it suggests substantial heterogeneity between centres. Also, the between-centre differences were expressed in a 95% range of odds ratios for each centre compared to the average centre [10].

Second, hospital volume was calculated for every MTC as the mean number of severely injured patients treated in one MTC per year. To assess the volume-outcome relationship, observed mortality rates were plotted against hospital volume for all MTCs. For the purpose of description of patient characteristics hospital volume was divided in tertiles.

Subsequently multivariable random effects logistic regression (in-hospital mortality) and linear regression (LOS, critical care LOS, time to first operation and time to first CT scan) models were used to analyse the effect of volume on outcome. Hospital volume was tested for nonlinearity using splines and Likelihood Ratio Test. Both the unadjusted and adjusted models contained hospital volume and a random intercept for centres. The adjusted models were based on clinically relevant confounders including age, sex, ISS, Revised Trauma Score (RTS), Charlson Comorbidity Index (CCI), penetrating injury, Abbreviated Injury Score (AIS) head injury and referral [11]. ISS was modelled with a spline function and an interaction term was added for the relationship between the effect of age and the effect of sex in accordance with the TARN model [12]. A sensitivity analysis included all patients directly transferred to a MTC. An extra sensitivity analysis was done using the new injury severity score (NISS) > 15 as criterium for severely injury, since NISS is more sensitive for head injury [13].

Statistical analyses were performed in R statistical software 3.4.2 (R Foundation for Statistical Computation, Vienna). Random effect models were fitted with Adaptive Gaussian Quadrature with 15 qpoints using the lme4 package.

## Results

### Descriptives

A total of 47,159 severely injured patients were included in this study. These patients were admitted to 28 MTCs, with volumes varying from 69 to 781 severely injured patients per year. Median age was 53 (Interquartile Range (IQR) 32–74), 70.1% of the patients were male and median ISS was 25 (17–29) (Table 1). The median Glasgow Coma Score (GCS) at the Emergency Department was 15 (IQR 14–15).

**Table 1:** Baseline characteristics

	Total *N* = 47,157	Tertile 1, volume ≤ 490, *N* = 16,280	Tertile 2, volume 491–574, *N* = 15,573	Tertile 3, volume > 574, *N* = 15,304
Number of MTCs	28	14	8	6
Age	53 (32–74)	56 (36–76)	52 (31–72)	53 (31–73)
Male	33,072 (70.1%)	11,224 (68.9%)	11,056 (71.0%)	10,792 (70.5%)
Penetrating injury	1364 (2.9%)	404 (2.5%)	543 (3.5%)	417 (2.7%)
ISS	25 (17–29)	25 (17–27)	25 (18–29)	25 (18–29)
NISS	34 (25–50)	34 (25–50)	34 (26–50)	34 (26–50)
GCS at arrival Emergency Department	15 (14–15)	15 (13–15)	15 (14–15)	15 (14–15)
Charlson Comorbidity Index	0 (0–3)	0 (0–4)	0 (0–3)	0 (0–4)
Intubation at Emergency Department	12,256 (26.0%)	3837 (23.6%)	4313 (27.7%)	4106 (26.8%)
Hypovolemic shock at Emergency Department (SBP < 90 mmHg)	8662 (18.4%)	2757 (16.9%)	3203 (20.6%)	2702 (17.7%)
AIS head ≥3	30,258 (64.2%)	10,409 (63.9%)	9822 (63.1%)	10,027 (65.5%)
RTS	7.84 (7.6–7.84)	7.8 (7.6–7.84)	7.8 (7.6–7.84)	7.8 (7.8–7.84)
Referred patients	15,118 (32.1%)	5194 (31.9%)	4577 (29.4%)	5347 (34.9%)
Length of Stay	10 (5–21)	10 (5–20)	11 (5–21)	10 (5–22)
Critical Care Length of Stay	0 (0–3)	0 (0–3)	0 (0–4)	0 (0–3)
In-hospital mortality	5876 (12.5%)	2047 (12.6%)	1937 (12.4%)	1892 (12.4%)

Continuous: median (IQR), categorical: N (%), New Injury Severity Score (NISS), Injury Severity Score (ISS), Abbreviated Injury Scale (AIS), Revised Trauma Score (RTS), Glasgow Coma Score (GSC)

In total 5876 patients died in-hospital (12.5%), the median LOS was 10 days (IQR 5–21) and the median critical care LOS was 0 days (IQR 0–3, Table 1). Volume was divided in tertiles (first tertile: hospital volume ≤ 490, *N* = 16,280, second tertile: hospital volume 491–574, *N* = 15,573, third tertile: hospital volume > 574, *N* = 15,304). There were no variation in baseline characteristics between the tertiles (Table 1).

### Between-Centre differences

**Fig. 1:**
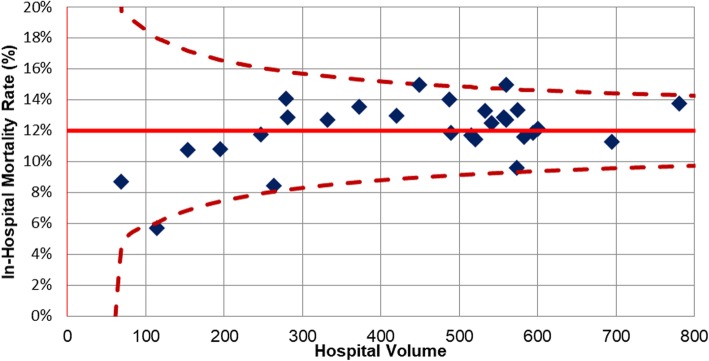
Forrest plot with observed mortality rates per MTC. Red line: fitted unadjusted linear regression model for the association between mortality rates and hospital volume with corresponding 95% Confidence Intervals

**Fig. 2:**
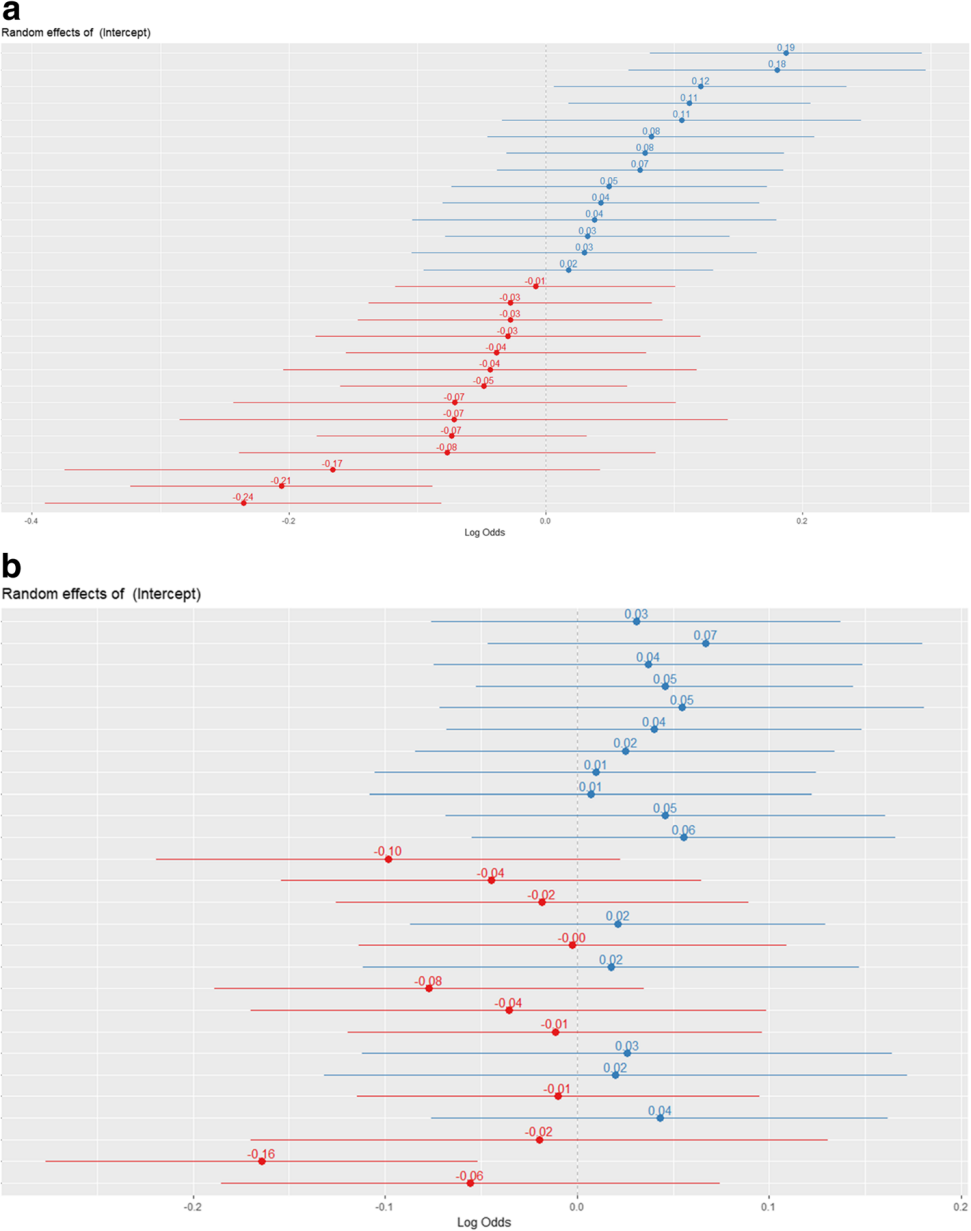
Differences in mortality rates between centres. Unadjusted differences between centers, log odds of 0 indicates average mortality, lines indicate 95% posterior interval. Differences between centers, adjusted for age, gender, age*gender, ISS (spline), RTS, Charlson Comorbidity Index, penetrating injury, AIS head injury and referred patients

The observed mortality rates varied from 4.7 to 15.0% (Fig. 1), but the random-effects model showed the true differences to be very small (in-hospital mortality tau-squared = 0.015). The 95% odds ratio range of centre effects was 0.97–1.03 (Table 2, Fig. 2a). After adjustment for patient characteristics, the between-centre differences decreased (tau-squared = 0.006) with a corresponding 95% range of centre effects of 0.99–1.01. This means that the odds of dying in the lowest percentile of centres (2.5th) was 0.99 times the average, while the odds of dying in the highest percentile of centres (97.5th) was 1.01 times the average (Fig. 2b).

**Table 2:** Between- centre differences for in-hospital mortality

	Tau^2^	95% centre Range
Unadjusted	0.015	0.97–1.03
Adjusted	0.006	0.99–1.01
Adjusted including volume	0.004	0.99–1.01

### Volume-outcome relationship: in-hospital mortality

There was a non-significant association between higher hospital volume and higher in-hospital mortality according to the unadjusted random effects model (OR 1.63 per 10 patients, 95% CI 0.98–2.71, *p* = 0.06, Table 3). After adjustment, there was no association between hospital volume and in-hospital mortality (OR 1.02, 95%CI 0.68–1.54, *p* = 0.92). Also, after excluding referred patients there was no significant association between hospital volume and in-hospital mortality (OR 0.71, 95% CI 0.41–1.22, *p* = 0.21). Hospital volume was considered linear (*p*-value of nonlinear term = 0.89), so no cut-off could be found. Using NISS > 15 as criterium for severely injured, found similar results (adjusted OR: 1.01, 95% 0.64–1.60, *p* = 0.96, Appendix).

**Table 3:** Unadjusted and adjusted coefficients of hospital volume for different outcome measures, expressed as odds ratio or beta per 10 patients

Outcome	OR per 10 patients	95% CI	P-value
**In-hospital mortality**			
Unadjusted OR	1.63	0.98–2.71	0.06
Adjusted* OR	1.02	0.68–1.54	0.92
Adjusted* OR excluding referred patients	0.71	0.41–1.22	0.21
	**Beta per 10 patients**		
**Length of stay (days)**			
Unadjusted β	0.05	−0.01-0.11	0.11
Adjusted* β	0.03	− 0.03-0.09	0.33
Adjusted* β excluding referred patients	0.07	0.00–0.14	0.06
**Critical care length of stay (days)**			
Unadjusted β	0.20	−0.25-0.65	0.39
Adjusted* β	−0.02	−2.84-2.80	0.93
Adjusted* β excluding referred patients	0.48	0.02–0.94	0.04
**Time to operation (hours)**			
Unadjusted β	−0.25	−0.70-0.20	0.28
Adjusted* β	−0.24	−0.70-0.22	0.31
Adjusted* β excluding referred patients	−0.41	−0.97-0.15	0.15
**Time to CT (hours)**			
Unadjusted β	−0.32	−0.61--0.03	0.03
Adjusted* β	−0.01	−0.02-0.004	0.16
Adjusted* β excluding referred patients	−0.03	− 0.08-0.02	0.22

*Adjusted for age, gender, age*gender (interaction term), ISS (spline), Revised Trauma Score, Charlson Comorbidity Index, penetrating injury, AIS head injury, referred patients (when not excluded)

### Volume-outcome relationship: secondary outcomes

There was no association between hospital volume and LOS, also after adjusting for patient characteristics (β = 0.03 per 10 patients, *p* = 0.33, Table 3). There was no association between hospital volume and critical care LOS after adjustment (β = − 0.61 per 10 patients, *p* = 0.78). After excluding referred patients, critical care LOS was associated with hospital volume (β = 0.48 per 10 patients, *p* = 0.04). In the adjusted models there was no association between hospital volume and time to first operation (adjusted β = − 0.24 per 10 patients, *p* = 0.31) or time to first CT scan (adjusted β = − 0.01 per 10 patients, *p* = 0.16).

## Discussion

This study aimed to evaluate whether there was an association between hospital volume and outcomes among severely injured patients in Major Trauma Centres. Despite the large variation in volume of the included MTCs, no relationship between hospital volume and outcome of severely injured patients was found, contrary to current beliefs [5]. Small between-centre differences for in-hospital mortality were found which suggests comparable outcomes between MTCs.

Centralization of care is suggested to improve cost-effectivity and patient outcomes [14, 15]. Most evidence for the benefit of regionalization in terms of hospital volume is found in elective surgical procedures [16–18]. It seems logical that severely injured patients could benefit from centralization, because severely injured patients often require complex care, having experience in treating those patients could improve patient outcomes. Over the past decades, centralization on trauma care, based on different criterions, took place showing beneficial outcomes [2, 19, 20]. MTCs have been established in England in 2012. A before-after study showed no significant improvements in mortality and LOS in the post-implementation analysis (270 days), although the caseload increased [3]. It is thought that benefits of regionalization will become visible over a number of years [21] when trauma services “mature” in terms of experience, pre-hospital triage and refinement of hospital systems [3, 22, 23]. A recent publication shows that the development of Major Trauma Networks including MTCs covering the entire national population increases the odds of survival for patients reaching the hospital alive [4]. This suggests that centralization without volume requirements shows beneficial results.

There are several explanations for small observed between-centre differences. First, TARN closely monitors MTCs with emphasis on outcomes. TARN provides hospitals with case-mix adjusted survival rates to help hospital clinicians to improve their system of trauma care. Second, MTCs need to fulfil various designation requirements which decreases variation in structure and processes. For example, MTCs must have 24/7 availability of consultants to lead the trauma team and 24/7 availability of fully staffed operating theatres. Also, MTCs are required to create a pathway from the prehospital phase to the rehabilitation phase for each severely injured patient [4]. To the best knowledge, no other study assessed inter-hospital variation for severely injured patients. Considerable between-centre differences have been found in the field of traumatic brain injury (TBI) [6–9], which were caused by structural differences between countries and centres. The current study showed no evidence for the volume-outcome relationship in severely injured patients treated in MTCs. This is in contrast with a recently published systematic review and meta-analysis which found a beneficial effect for high volume centres [5]. However, most of these studies included both MTCs and non MTCs, so a potential volume effect could be biased by other factors. A further consideration, most of these studies were performed in the United States which differs in terms of geography, infrastructure and trauma epidemiology compared to the England. England has more densely populated areas, shorter transportation distances, and the already existing infrastructure of district general hospitals providing universal acute care coverage [24, 25]. The designation criteria for MTCs do not include a hospital volume requirement, so hospital volume differed from 69 to 781 severely injured patients per year [26]. Therefore, it was possible to assess hospital volume in a linear rather than categorical fashion which provided a more in-depth assessment of centre effects.

Increasing hospital volume was associated with a longer critical care LOS after excluding referred patients. There was no association between hospital volume and critical care LOS when including all severely injured patients. The most evident explanation for the association between hospital volume and critical care LOS is chance. It is also possible that referred patients come after they stayed at the ICU at their referring hospital and therefore have shorter LOS.

Other factors than hospital volume cause the extremely small between-centre differences in MTCs. The most evident explanation is differences in patient characteristics. After adjusting for several demographic and injury severity characteristics, higher hospital volume was not associated with lower mortality. A limitation of this study is that insufficient adjustment of case-mix differences is possible. With use of the TARN model [12] extended with clinically relevant variables from the TRISS model, adjustments for case-mix differences between MTCs were made. However, the risk of residual confounding cannot be exclude. Also, the results might be influenced by a few very well organized MTCs. It was not possible to assess the relationship between surgeon volume and outcomes. Other studies that investigated this relationship showed inconsistent results [5, 27–29]. The caseload and experience per surgeon might influence between-centre differences. Also, we were unable to assess the health care provider - patient ratio and Critical Care volume bed - availability ratio. Our results are only applicable to MTCs and can thereforenot be generalized to non MTCs with low volumes of severely injured patients. Also, the prehospital network is important for the outcomes of severely injured patients. Detailed prehospital data was not available when doing this study. In order to investigate whether these results can be extrapolated to other trauma systems, it is important to take the prehospital systems into account. Another limitation is the lack of a good definition of the severely injured patient. The universally used injury severity measure in trauma registries and research is ISS, where ISS > 15 is defined as severely injured. However, questions about the accuracy of ISS have been raised. First, an equal Abbreviated Injury Scale (AIS) in different body regions is assumed to be equal in injury severity [30, 31]. Second, ISS does not account for multiple injuries in the same body region [31, 32]. So it is possible that patients with equal ISS scores do not have the same injury severity. Therefore, future research should examine which patient groups really benefit from treatment at a MTC, to make optimal use of the resources and expertise of MTCs. A sensitivity analysis using the NISS > 15 as severely injured showed no association between hospital volume and outcomes in MTCs.

## Conclusions

Despite a tenfold variation in volume, no differences in outcomes of severely injured patients were found between English MTCs. These results suggest that MTCs in England achieve comparable outcomes in severely injured patients despite the number of severely injured patients they treat. Centres with similar structure and processes of care can achieve comparable outcomes for severely injured patients. Further research is necessary to see whether these results can be extrapolated to trauma systems in other countries.

## Appendix

### Analyses with NISS> 15 instead of ISS > 15

**Table 4:** Baseline characteristics

	Total *N* = 60,146
Age	53 (33–74)
Male	41,435 (68.9%)
Penetrating injury	2235 (3.7%)
ISS	21 (16–26)
NISS	29 (19–40)
GCS at arrival Emergency Department	15 (13–15)
Charlson Comorbidity Index	0 (0–3)
Intubation at Emergency Department	12,961 (21.5%)
Hypovolemic shock at Emergency Department (SBP < 90 mmHg)	10,667 (17.7%)
AIS head ≥3	27,850 (46.3%)
RTS	7.84 (7.7–7.84)
Referred patients	18,151 (30.1%)

Continuous: median (IQR), categorical: N (%), New Injury Severity Score (NISS), Injury Severity Score (ISS), Abbreviated Injury Scale (AIS), Revised Trauma Score (RTS), Glasgow Coma Score (GSC)

**Table 5:** Unadjusted and adjusted coefficients of hospital volume for different outcome measures, expressed as odds ratio or beta

Outcome	OR per 10 patients	95% CI	P-value
**In-hospital mortality**			
Unadjusted OR	1.64	1.04–2.61	0.03
Adjusted* OR	1.01	0.64–1.60	0.96
	**Beta**		
**Length of stay (days)**			
Unadjusted β	0.66	−0.15 - 1.47	0.11
Adjusted* β	0.40	− 0.45 - 1.24	0.34
**Critical care length of stay (days)**			
Unadjusted β	−0.02	−0.36 – 0.32	0.89
Adjusted* β	−0.04	−0.32 – 0.23	0.76
**Time to operation (hours)**			
Unadjusted β	0.004	−0.03 - 0.03	0.97
Adjusted* β	0.005	−0.03 – 0.03	0.75
**Time to CT (hours)**			
Unadjusted β	−0.03	−0.05 - -0.01	0.01
Adjusted* β	−0.003	−0.007 - 0.001	0.11

*Adjusted for age, gender, age*gender (interaction term), NISS (spline), Revised Trauma Score, Charlson Comorbidity Index, penetrating injury, referred patients

## Abbreviations

AIS: Abbreviated Injury Scale
AOR: Adjusted Odds Ratio
CCI: Charlson Comorbidity Index
ISS: Injury Severity Score
LOS: Length of stay
MTC: Major Trauma Centre
OR: Odds Ratio
RTS: Revised Trauma Score
TARN: Trauma Audit and Research Network
TBI: Traumatic Brain Injury

## References

[1] Polinder S, Haagsma JA, Toet H, van Beeck EF (2012). Epidemiological burden of minor, major and fatal trauma in a national injury pyramid. Br J Surg.

[2] Celso B, Tepas J, Langland-Orban B, Pracht E, Papa L, Lottenberg L (2006). A systematic review and meta-analysis comparing outcome of severely injured patients treated in trauma centers following the establishment of trauma systems. J Trauma.

[3] Metcalfe D, Perry DC, Bouamra O, Salim A, Woodford M, Edwards A (2016). Regionalisation of trauma care in England. Bone Joint J.

[4] Moran CG, Lecky F, Bouamra O, Lawrence T, Edwards A, Woodford M (2018). Changing the system-major trauma patients and their outcomes in the NHS (England) 2008–17. EClinicalMedicine..

[5] Sewalt CA, Wiegers EJA, Venema E, Lecky FE, Schuit SCE, Hartog DD (2018). The volume-outcome relationship in severely injured patients: a systematic review and meta-analysis. J Trauma Acute Care Surg.

[6] Clifton GL, Choi SC, Miller ER, Levin HS, Smith KR, Muizelaar JP (2001). Intercenter variance in clinical trials of head trauma--experience of the National Acute Brain Injury Study: hypothermia. J Neurosurg.

[7] Clifton GL, Drever P, Valadka A, Zygun D, Okonkwo D (2009). Multicenter trial of early hypothermia in severe brain injury. J Neurotrauma.

[8] Lingsma HF, Roozenbeek B, Li B, Lu J, Weir J, Butcher I (2011). Large between-center differences in outcome after moderate and severe traumatic brain injury in the international mission on prognosis and clinical trial design in traumatic brain injury (IMPACT) study. Neurosurgery..

[9] Maas AI, Murray G, Henney H, Kassem N, Legrand V, Mangelus M (2006). Efficacy and safety of dexanabinol in severe traumatic brain injury: results of a phase III randomised, placebo-controlled, clinical trial. Lancet Neurol.

[10] Timbie JW, Normand SL (2008). A comparison of methods for combining quality and efficiency performance measures: profiling the value of hospital care following acute myocardial infarction. Stat Med.

[11] Schluter PJ (2011). The trauma and injury severity score (TRISS) revised. Injury..

[12] Bouamra O, Jacques R, Edwards A, Yates DW, Lawrence T, Jenks T (2015). Prediction modelling for trauma using comorbidity and ‘true’ 30-day outcome. Emerg Med J.

[13] Samin OA, Civil ID. The new injury severity score versus the injury severity score in predicting patient outcome: a comparative evaluation on trauma service patients of the Auckland hospital. P Ann C Ass. 1999;43:1–15.

[14] Gordon TA, Burleyson GP, Tielsch JM, Cameron JL (1995). The effects of regionalization on cost and outcome for one general high-risk surgical procedure. Ann Surg.

[15] Pollack MM, Alexander SR, Clarke N, Ruttimann UE, Tesselaar HM, Bachulis AC (1991). Improved outcomes from tertiary center pediatric intensive care: a statewide comparison of tertiary and nontertiary care facilities. Crit Care Med.

[16] Begg CB, Cramer LD, Hoskins WJ, Brennan MF (1998). Impact of hospital volume on operative mortality for major cancer surgery. JAMA..

[17] Birkmeyer JD, Finlayson SR, Tosteson AN, Sharp SM, Warshaw AL, Fisher ES (1999). Effect of hospital volume on in-hospital mortality with pancreaticoduodenectomy. Surgery..

[18] Ellison LM, Heaney JA, Birkmeyer JD (2000). The effect of hospital volume on mortality and resource use after radical prostatectomy. J Urol.

[19] MacKenzie EJ, Morris JA, Smith GS, Fahey M (1990). Acute hospital costs of trauma in the United States: implications for regionalized systems of care. J Trauma.

[20] Sampalis JS, Denis R, Lavoie A, Frechette P, Boukas S, Nikolis A (1999). Trauma care regionalization: a process-outcome evaluation. J Trauma.

[21] Mullins RJ, Veum-Stone J, Helfand M, Zimmer-Gembeck M, Hedges JR, Southard PA (1994). Outcome of hospitalized injured patients after institution of a trauma system in an urban area. JAMA..

[22] Barquist E, Pizzutiello M, Tian L, Cox C, Bessey PQ (2000). Effect of trauma system maturation on mortality rates in patients with blunt injuries in the Finger Lakes region of New York state. J Trauma.

[23] Peitzman AB, Courcoulas AP, Stinson C, Udekwu AO, Billiar TR, Harbrecht BG (1999). Trauma center maturation: quantification of process and outcome. Ann Surg.

[24] Nicholl J, Turner J (1997). Effectiveness of a regional trauma system in reducing mortality from major trauma: before and after study. BMJ..

[25] Kanakaris NK, Giannoudis PV (2011). Trauma networks: present and future challenges. BMC Med.

[26] England N. NHS standard contract for major trauma service (all ages). England N: NHS Commissioning Board. 2014.

[27] Konvolinka CW, Copes WS, Sacco WJ (1995). Institution and per-surgeon volume versus survival outcome in Pennsylvania's trauma centers. Am J Surg.

[28] Margulies DR, Cryer HG, McArthur DL, Lee SS, Bongard FS, Fleming AW (2001). Patient volume per surgeon does not predict survival in adult level I trauma centers. J Trauma.

[29] Sava J, Kennedy S, Jordan M, Wang D (2003). Does volume matter? The effect of trauma surgeons’ caseload on mortality. J Trauma.

[30] Chawda MN, Hildebrand F, Pape HC, Giannoudis PV (2004). Predicting outcome after multiple trauma: which scoring system?. Injury..

[31] Koksal O, Ozdemir F, Bulut M, Aydin S, Almacioglu ML, Ozguc H (2009). Comparison of trauma scoring systems for predicting mortality in firearm injuries. Ulus Travma Acil Cerrahi Derg.

[32] Tamim H, Al Hazzouri AZ, Mahfoud Z, Atoui M, El-Chemaly S (2008). The injury severity score or the new injury severity score for predicting mortality, intensive care unit admission and length of hospital stay: experience from a university hospital in a developing country. Injury..

